# Supplementary material for: Does who I am and what I feel determine what I see (or say)? A meta-analytic systematic review exploring the influence of real and perceived bodily state on spatial perception of the external environment
Source: PeerJ. 2022 May 23;10:e13383. doi: 10.7717/peerj.13383 (PMC9135041; doi:10.7717/peerj.13383)
Supplement: Supplemental Information 1 [file peerj-10-13383-s001.docx]

| Supplementary 1: Inclusion and exclusion criteria in PICOS format | | |
| --- | --- | --- |
|  | Inclusion | Exclusion |
| **Population** | Adults (≥18 years of age), both healthy and clinical populations | Mixed age populations, unless ≥80% of the sample were adults or if data were presented separately for adults versus children |
| **Intervention** | Bodily states, both stable and fluctuating, that are related to physiological (e.g., blood glucose) or morphological (e.g., body size/weight) features  Perceptions of bodily state (perceived morphology or capacity) | Bodily states related to emotion, social or affect  Studies related to tools/tool use (i.e., where the aim is to manipulate peripersonal space) |
| **Comparators** | 1. Groups of participants with differing bodily states, or 2. Manipulation of bodily state within a group of participants 3. No comparator but evaluates an association between bodily state and spatial perception. | The only comparator group or condition involves emotion/affect or tool use. |
| **Outcomes** | Environmental spatial perception measures that  quantitatively evaluate the perception of extra-personal space (e.g., distance measure, hill steepness perception). | Measures of spatial perception of the peri-personal space, a body part, or an object, or qualitative assessments of spatial perception.  Surrogate measures of spatial perception (i.e., not directly asked about spatial perception) |
| **Study design** | Designs (experimental or clinical trial) that use within and/or between group comparisons. | Case studies |
