# Supplementary material for: Does who I am and what I feel determine what I see (or say)? A meta-analytic systematic review exploring the influence of real and perceived bodily state on spatial perception of the external environment
Source: PeerJ. 2022 May 23;10:e13383. doi: 10.7717/peerj.13383 (PMC9135041; doi:10.7717/peerj.13383)
Supplement: Supplemental Information 2 [file peerj-10-13383-s002.docx]

Supplementary 2: Full search strategy

Medline (OVID) search strategy:

| 1 | Space Perception/ |
| --- | --- |
| 2 | Distance Perception/ |
| 3 | Visual Perception/ |
| 4 | (((perceiv* or percept*) adj2 (spatial or space or distance)) or ((slant or steep or steepness) adj4 (hill? or stair*))).tw,kf. |
| 5 | or/1-4 |
| 6 | Physiology/ |
| 7 | Obesity/ |
| 8 | Body Weight/ |
| 9 | Body Mass Index/ |
| 10 | Body Size/ |
| 11 | Body Constitution/ |
| 12 | Body Composition/ |
| 13 | Body Fat Distribution/ |
| 14 | Self concept/ |
| 15 | Body Image/ |
| 16 | Pain/ |
| 17 | Chronic pain/ |
| 18 | Fatigue/ |
| 19 | Physical fitness/ |
| 20 | Blood Glucose/ |
| 21 | Energy metabolism/ |
| 22 | (obes* or overweight or ((body or bodily or self) adj3 (state* or perceiv* or perception* or concept*)) or body weight* or bodyweight* or adiposity or intractable pain or chronic pain or persistent pain or blood glucose or bioenergetic state or energetic state or fitness or action capabilities or fatigue or body image).tw,kf. |
| 23 | or/6-22 |
| 24 | 5 and 23 |

PsychINFO (OVID) search strategy:

| 1 | Spatial Perception/ |
| --- | --- |
| 2 | Distance Perception/ |
| 3 | Visual Perception/ |
| 4 | (((perceiv* or percept*) adj2 (spatial or space or distance)) or ((slant or steep or steepness) adj4 (hill? or stair*))).tw |
| 5 | or/1-4 |
| 6 | Physiology/ |
| 7 | Obesity/ |
| 8 | Body Weight/ |
| 9 | Body Mass Index/ |
| 10 | Body Size/ |
| 11 | Body Image/ |
| 12 | Self Perception/ |
| 13 | Self concept/ |
| 14 | Body Image/ |
| 15 | Pain/ |
| 16 | Chronic pain/ |
| 17 | Fatigue/ |
| 18 | Physical fitness/ |
| 19 | Blood Glucose/ |
| 20 | Energy expenditure/ |
| 21 | (obes* or overweight or ((body or bodily or self) adj3 (state* or perceiv* or perception* or concept*)) or body weight* or bodyweight* or adiposity or intractable pain or chronic pain or persistent pain or blood glucose or bioenergetic state or energetic state or fitness or action capabilities or fatigue or body image).tw |
| 22 | or/6-21 |
| 23 | 5 and 22 |

Embase (OVID) search strategy

| 1 | Depth Perception/ |
| --- | --- |
| 2 | Distance Perception/ |
| 3 | (((perceiv* or percept*) adj2 (spatial or space or distance)) or ((slant or steep or steepness) adj4 (hill? or stair*))).tw,kw |
| 4 | or/1-3 |
| 5 | Exercise physiology/ |
| 6 | Obesity/ |
| 7 | Body Mass Index/ |
| 8 | Body Size/ |
| 9 | Body condition/ |
| 10 | Body constitution/ |
| 11 | Body Fat Distribution/ |
| 12 | Self concept/ |
| 13 | Body Image/ |
| 14 | Pain/ |
| 15 | Chronic pain/ |
| 16 | Fatigue/ |
| 17 | Exhaustion/ |
| 18 | Fitness/ |
| 19 | Blood Glucose Level/ |
| 20 | Energy metabolism/ |
| 21 | Energy balance/ |
| 22 | (obes* or overweight or ((body or bodily or self) adj3 (state* or perceiv* or perception* or concept*)) or body weight* or bodyweight* or adiposity or intractable pain or chronic pain or persistent pain or blood glucose or bioenergetic state or energetic state or fitness or action capabilities or fatigue or body image). Tw,kw |
| 23 | or/5-22 |
| 24 | 4 and 23 |

Emcare (OVID) search strategy:

| 1 | Depth Perception/ |
| --- | --- |
| 2 | Distance Perception/ |
| 3 | (((perceiv* or percept*) adj2 (spatial or space or distance)) or ((slant or steep or steepness) adj4 (hill? or stair*))).ab,ti,kw |
| 4 | or/1-3 |
| 5 | Exercise physiology/ |
| 6 | Obesity/ |
| 7 | Body Mass Index/ |
| 8 | Body Size/ |
| 9 | Body condition/ |
| 10 | Body constitution/ |
| 11 | Body Fat Distribution/ |
| 12 | Self concept/ |
| 13 | Body Image/ |
| 14 | Pain/ |
| 15 | Chronic pain/ |
| 16 | Fatigue/ |
| 17 | Exhaustion/ |
| 18 | Fitness/ |
| 19 | Blood Glucose Level/ |
| 20 | Energy metabolism/ |
| 21 | Energy balance/ |
| 22 | (obes* or overweight or ((body or bodily or self) adj3 (state* or perceiv* or perception* or concept*)) or body weight* or bodyweight* or adiposity or intractable pain or chronic pain or persistent pain or blood glucose or bioenergetic state or energetic state or fitness or action capabilities or fatigue or body image). ab,ti,kw |
| 23 | or/5-22 |
| 24 | 4 and 23 |

Scopus (Elsevier) search strategy:

( TITLE-ABS-KEY ( ( ( perceiv*  OR  percept* )  W/1  ( spatial  OR  space  OR  distance ) )  OR  ( ( slant  OR  steep  OR  steepness )  W/3  ( hill  OR  hills  OR  stair* ) ) ) )  AND  ( TITLE-ABS-KEY ( obes*  OR  overweight  OR  "body weight*"  OR  bodyweight*  OR  adiposity  OR  "intractable pain"  OR  "persistent pain"  OR  "chronic pain"  OR  "blood glucose"  OR  "bioenergetic state"  OR  "energetic state"  OR  fitness  OR  fatigue  OR  "body image"  OR  ( ( body  OR  bodily  OR  self )  W/2  ( state*  OR  perceiv*  OR  perception*  OR  concept* ) ) ) )

Conference Abstracts

The following conferences will be searched for relevant abstracts from 2010-2020.

- Vision Sciences Society
- European Conference on Visual Perception

ProQuest Dissertations and Theses Global:

(( perceiv*  OR  percept* )  n/1  ( spatial  OR  space  OR  distance ))  OR  (( slant  OR  steep  OR  steepness )  n/3  ( hill  OR  hills  OR  stair* ) )

AND

(obes*  OR  overweight  OR  "body weight*"  OR  bodyweight*  OR  adiposity  OR  "intractable pain"  OR  "persistent pain"  OR  "chronic pain"  OR  "blood glucose"  OR  "bioenergetic state"  OR  "energetic state"  OR  fitness  OR “action capabilities”  fatigue  OR  "body image"  OR  ( ( body  OR  bodily  OR  self )  n/2  ( state*  OR  perceiv*  OR  perception*  OR  concept* ) ) )

Google Scholar search strategy:

| **#** | **Search Terms** | **Results Screened** |
| --- | --- | --- |
| 1 | spatial perception OR distance perception OR visual perception AND body state | 20 |
| 2 | spatial perception OR distance perception OR visual perception AND economy of action | 20 |
| 3 | spatial perception OR distance perception OR visual perception AND bioenergetics | 20 |
| 4 | spatial perception OR distance perception OR visual perception AND embodied perception | 20 |
| 5 | Spatial perception AND body state | 20 |
| 6 | Distance perception AND body state | 20 |
| 7 | Visual perception AND body state | 20 |
| 8 | Spatial perception AND perceived body state | 20 |
| 9 | Distance perception AND perceived body state | 20 |
| 10 | Visual perception AND perceived body state | 20 |
