# Supplementary material for: Does who I am and what I feel determine what I see (or say)? A meta-analytic systematic review exploring the influence of real and perceived bodily state on spatial perception of the external environment
Source: PeerJ. 2022 May 23;10:e13383. doi: 10.7717/peerj.13383 (PMC9135041; doi:10.7717/peerj.13383)
Supplement: Supplemental Information 3 [file peerj-10-13383-s003.docx]

Supplementary 3: Risk of Bias form and grading instructions

| Questions | Bias Judgement instructions: |
| --- | --- |
| How were participants selected? Convenience or random sampling? | Low = Random sampling  High = Non-random sampling (e.g., convience sampling)  Unsure = Not reported |
| Was sample size justification, power description, or variance and effect estimates provided? And was the target sample size achieved? | Low = if a priori calculation done and justified, reported and achieved.  High = if a priori calculation inadequate, or done but sample size not achieved  Unsure = Not reported  N.A. = |
| Was group allocation (or condition order for within subject designs) randomised? | Low = Subjects randomised to groups adequately  High = insufficient method Unsure = Not reported  N.A. = For studies where this is not required (e.g. within group studies) |
| Was the spatial perception task randomised? | Low = Spatial perception task order randomized and adequate (e.g., distances randomized, estimation type randomized)  High = insufficient method Unsure = Not reported |
| Were key potential confounding variables measured and controlled for either methodologically or statistically for their impact on the relationship between exposure(s) and outcome(s)? | Potential confounding factors: age, gender, fatigue, body weight, pain. This is not an exhaustive list NOR do all of these have to be controlled for - use your judgement based on the studies design and research question  Low = Confounding measures quantified and controlled for  High = Confounding measures discussed but not controlled for  Unsure = Not reported |
| For within group studies, was the intended manipulation of bodily state achieved and was this measured? For between group studies was the bodily state measured in both groups and were they different? | Low = Measured the body state across all groups and timepoints, and this measurement was quantified.  High = Measured, but not in a quantifiable manner or not at all time points or groups.  Unsure = Not reported |
| Was the reliability of the spatial perception measure assessed? | Low = Reported, referenced, and similar to referenced paper (e.g., similar distance/slope)  High = Reported but not similar to the referenced paper.  Unsure = Not reported |
| Were participants blinded to the nature of the study? Was this blinding credible? | Low = Reported and blinding was assessed to be credible (e.g, post-experiment questionnaires)  High = Reported but the blinding does not seem credible and this was not assessed.  Unsure = Not reported |
| Were assessors blinded to the exposure status (body state) of participants? | Low = Reported and possible  High = Not reported but based on the information provided able to make a judgement as to whether blinding is impossible to do (eg - young vs. old), or reported but the unlikely to be truely blind Unsure = Not reported and unable to make a determination based on the information provided |
| Was the analysis completed by an independent blinded researcher? | Low = Reported and possible  High = Reported but unlikely to be true Unsure = Not reported |
| Was the hypothesis determined a priori? | Low = Reported and able to confirm that this is truely a priori (eg - trial registration)  High = Have a hypothesis in the paper, but unable to determine if a priori  Unsure = Not reported |
| Was the analysis determined a priori? | Low = Reported and this plan was followed. If the plan was not followed then ammendments must be justified and appropriate. This includes pre-registation of the trial.  High = Yes, but the analysis was different than this plan. Unsure = Not reported |
| Were all measured outcomes data reported? | Low = All outcomes discussed in the methods are reported in the results section  High = Results are not reported for some of the outcomes  Unsure = We are not sure that all results are reported |
| Missing data <15%? | Consider that all data missing from analysis is missing (eg, drop outs, outliers that are excluded) Low = Missing data less than 15%  High = Missing data greater than 15%  Unsure = Not reported |
| Other |  |
