# Supplementary material for: Does who I am and what I feel determine what I see (or say)? A meta-analytic systematic review exploring the influence of real and perceived bodily state on spatial perception of the external environment
Source: PeerJ. 2022 May 23;10:e13383. doi: 10.7717/peerj.13383 (PMC9135041; doi:10.7717/peerj.13383)
Supplement: Supplemental Information 5 [file peerj-10-13383-s005.docx]

Supplementary 5: Sensitivity analysis for correlation coefficient for within-group studies.

Table 1: Verbal distance estimation for normal sleep, pre- and post-exercise

|  | Paper | Measure | Hedge's g | Variance | SE | LL CI | UL CI |
| --- | --- | --- | --- | --- | --- | --- | --- |
| r=0.262 | Baati et al., 2020 | 15m (NNS pre and post exercise) | -0.022 | 0.123 | 0.351 | -0.711 | 0.667 |
|  | Baati *et al.,* 2015 | 15m (NNS pre and post exercise) | -0.117 | 0.124 | 0.353 | -0.809 | 0.574 |
|  | Baati et al., 2020 | 25m (NNS pre and post exercise) | 0.080 | 0.124 | 0.352 | -0.610 | 0.770 |
|  | Baati *et al.,* 2015 | 25m (NNS pre and post exercise) | -0.527 | 0.144 | 0.379 | -1.271 | 0.216 |
|  | Baati et al., 2020 | 35m (NNS pre and post exercise) | -0.192 | 0.126 | 0.355 | -0.888 | 0.504 |
|  | Baati *et al.,* 2015 | 35m (NNS pre and post exercise) | -0.191 | 0.126 | 0.355 | -0.887 | 0.505 |
| r=0.362 | Baati et al., 2020 | 15m (NNS pre and post exercise) | -0.022 | 0.107 | 0.327 | -0.662 | 0.618 |
|  | Baati *et al.,* 2015 | 15m (NNS pre and post exercise) | -0.116 | 0.108 | 0.328 | -0.759 | 0.526 |
|  | Baati et al., 2020 | 25m (NNS pre and post exercise) | 0.080 | 0.107 | 0.327 | -0.561 | 0.721 |
|  | Baati *et al.,* 2015 | 25m (NNS pre and post exercise) | -0.523 | 0.124 | 0.352 | -1.213 | 0.168 |
|  | Baati et al., 2020 | 35m (NNS pre and post exercise) | -0.191 | 0.109 | 0.330 | -0.838 | 0.456 |
|  | Baati *et al.,* 2015 | 35m (NNS pre and post exercise) | -0.190 | 0.109 | 0.330 | -0.837 | 0.457 |
| r=0.162 | Baati et al., 2020 | 15m (NNS pre and post exercise) | -0.022 | 0.140 | 0.374 | -0.756 | 0.712 |
|  | Baati *et al.,* 2015 | 15m (NNS pre and post exercise) | -0.118 | 0.141 | 0.376 | -0.855 | 0.619 |
|  | Baati et al., 2020 | 25m (NNS pre and post exercise) | 0.080 | 0.141 | 0.375 | -0.655 | 0.815 |
|  | Baati *et al.,* 2015 | 25m (NNS pre and post exercise) | -0.531 | 0.164 | 0.405 | -1.324 | 0.262 |
|  | Baati et al., 2020 | 35m (NNS pre and post exercise) | -0.193 | 0.143 | 0.378 | -0.935 | 0.549 |
|  | Baati *et al.,* 2015 | 35m (NNS pre and post exercise) | -0.192 | 0.143 | 0.378 | -0.934 | 0.549 |

Table 2: Verbal distance estimation for normal sleep, pre- and post-exercise

|  | Paper | Measure | Hedge's g | Variance | SE | LL CI | UL CI |
| --- | --- | --- | --- | --- | --- | --- | --- |
| r=0.262 | Baati et al., 2020 | 15m (1st half sleep deprivation [SDB] pre and post exercise) | 0.068 | 0.124 | 0.352 | -0.622 | 0.757 |
|  | Baati et al., 2020 | 15m (2nd half sleep deprivation [SDE] pre and post exercise) | 0.301 | 0.130 | 0.361 | -0.406 | 1.008 |
|  | Baati *et al.,* 2015 | 15m (Total sleep deprivation pre and post exercise) | 0.073 | 0.124 | 0.352 | -0.616 | 0.763 |
|  | Baati et al., 2020 | 25m (1st half sleep deprivation [SDB] pre and post exercise) | -0.134 | 0.125 | 0.353 | -0.826 | 0.558 |
|  | Baati et al., 2020 | 25m (2nd half sleep deprivation [SDE] pre and post exercise) | -0.353 | 0.133 | 0.364 | -1.067 | 0.360 |
|  | Baati *et al.,* 2015 | 25m (Total sleep deprivation pre and post exercise) | -0.241 | 0.128 | 0.357 | -0.942 | 0.459 |
|  | Baati et al., 2020 | 35m (1st half sleep deprivation [SDB] pre and post exercise) | -0.097 | 0.124 | 0.352 | -0.787 | 0.594 |
|  | Baati et al., 2020 | 35m (2nd half sleep deprivation [SDE] pre and post exercise) | -0.067 | 0.124 | 0.352 | -0.756 | 0.622 |
|  | Baati *et al.,* 2015 | 35m (Total sleep deprivation pre and post exercise) | 0.091 | 0.124 | 0.352 | -0.599 | 0.781 |
| r=0.362 | Baati et al., 2020 | 15m (1st half sleep deprivation [SDB] pre and post exercise) | 0.067 | 0.107 | 0.327 | -0.574 | 0.708 |
|  | Baati et al., 2020 | 15m (2nd half sleep deprivation [SDE] pre and post exercise) | 0.297 | 0.112 | 0.335 | -0.360 | 0.954 |
|  | Baati *et al.,* 2015 | 15m (Total sleep deprivation pre and post exercise) | 0.073 | 0.107 | 0.327 | -0.568 | 0.714 |
|  | Baati et al., 2020 | 25m (1st half sleep deprivation [SDB] pre and post exercise) | -0.134 | 0.108 | 0.328 | -0.778 | 0.510 |
|  | Baati et al., 2020 | 25m (2nd half sleep deprivation [SDE] pre and post exercise) | -0.353 | 0.115 | 0.339 | -1.017 | 0.310 |
|  | Baati *et al.,* 2015 | 25m (Total sleep deprivation pre and post exercise) | -0.241 | 0.110 | 0.332 | -0.892 | 0.410 |
|  | Baati et al., 2020 | 35m (1st half sleep deprivation [SDB] pre and post exercise) | -0.097 | 0.107 | 0.328 | -0.738 | 0.545 |
|  | Baati et al., 2020 | 35m (2nd half sleep deprivation [SDE] pre and post exercise) | -0.067 | 0.107 | 0.327 | -0.708 | 0.574 |
|  | Baati *et al.,* 2015 | 35m (Total sleep deprivation pre and post exercise) | 0.091 | 0.107 | 0.327 | -0.551 | 0.732 |
| r=0.162 | Baati et al., 2020 | 15m (1st half sleep deprivation [SDB] pre and post exercise) | 0.068 | 0.140 | 0.375 | -0.667 | 0.802 |
|  | Baati et al., 2020 | 15m (2nd half sleep deprivation [SDE] pre and post exercise) | 0.304 | 0.148 | 0.385 | -0.450 | 1.058 |
|  | Baati *et al.,* 2015 | 15m (Total sleep deprivation pre and post exercise) | 0.074 | 0.141 | 0.375 | -0.661 | 0.808 |
|  | Baati et al., 2020 | 25m (1st half sleep deprivation [SDB] pre and post exercise) | -0.134 | 0.142 | 0.376 | -0.872 | 0.603 |
|  | Baati et al., 2020 | 25m (2nd half sleep deprivation [SDE] pre and post exercise) | -0.354 | 0.151 | 0.388 | -1.114 | 0.407 |
|  | Baati *et al.,* 2015 | 25m (Total sleep deprivation pre and post exercise) | -0.242 | 0.145 | 0.381 | -0.988 | 0.505 |
|  | Baati et al., 2020 | 35m (1st half sleep deprivation [SDB] pre and post exercise) | -0.097 | 0.141 | 0.375 | -0.832 | 0.639 |
|  | Baati et al., 2020 | 35m (2nd half sleep deprivation [SDE] pre and post exercise) | -0.067 | 0.140 | 0.375 | -0.802 | 0.668 |
|  | Baati *et al.,* 2015 | 35m (Total sleep deprivation pre and post exercise) | 0.091 | 0.141 | 0.375 | -0.645 | 0.826 |

Table 3:

|  | Paper | Measure | Hedge's g | Varience | SE | LL CI | UL CI |
| --- | --- | --- | --- | --- | --- | --- | --- |
| r=0.262 | Baati et al., 2020 | 15m (normal vs. first half of sleep deprivation) | 0.362 | 0.133 | 0.365 | -0.353 | 1.077 |
|  | Baati et al., 2020 | 25m (normal vs. first half of sleep deprivation) | 0.295 | 0.130 | 0.360 | -0.411 | 1.001 |
|  | Baati et al., 2020 | 35m (normal vs. first half of sleep deprivation) | 0.904 | 0.184 | 0.429 | 0.064 | 1.744 |
|  | Baati et al., 2020 | 15m (normal vs. second half of sleep deprivation) | 0.394 | 0.135 | 0.367 | -0.326 | 1.113 |
|  | Baati et al., 2020 | 25m (normal vs. second half of sleep deprivation) | 0.289 | 0.130 | 0.360 | -0.416 | 0.995 |
|  | Baati et al., 2020 | 35m (normal vs. second half of sleep deprivation) | 0.772 | 0.167 | 0.409 | -0.030 | 1.574 |
|  | Baati et al., 2015 | 15m (normal vs. sleep deprivation) | 0.438 | 0.138 | 0.371 | -0.289 | 1.165 |
|  | Baati *et al.,* 2015 | 25m (normal vs. sleep deprivation) | 0.627 | 0.152 | 0.390 | -0.138 | 1.392 |
|  | Baati *et al.,* 2015 | 35m (normal vs. sleep deprivation) | 0.651 | 0.155 | 0.393 | -0.120 | 1.422 |
| r=0.162 | Baati et al., 2020 | 15m (normal vs. first half of sleep deprivation) | 0.365 | 0.151 | 0.389 | -0.397 | 1.127 |
|  | Baati et al., 2020 | 25m (normal vs. first half of sleep deprivation) | 0.295 | 0.147 | 0.384 | -0.457 | 1.048 |
|  | Baati et al., 2020 | 35m (normal vs. first half of sleep deprivation) | 0.905 | 0.209 | 0.457 | 0.010 | 1.801 |
|  | Baati et al., 2020 | 15m (normal vs. second half of sleep deprivation) | 0.401 | 0.154 | 0.392 | -0.367 | 1.169 |
|  | Baati et al., 2020 | 25m (normal vs. second half of sleep deprivation) | 0.289 | 0.147 | 0.384 | -0.462 | 1.041 |
|  | Baati et al., 2020 | 35m (normal vs. second half of sleep deprivation) | 0.776 | 0.191 | 0.437 | -0.079 | 1.632 |
|  | Baati et al., 2015 | 15m (normal vs. sleep deprivation) | 0.451 | 0.157 | 0.396 | -0.326 | 1.228 |
|  | Baati *et al.,* 2015 | 25m (normal vs. sleep deprivation) | 0.628 | 0.173 | 0.416 | -0.188 | 1.443 |
|  | Baati *et al.,* 2015 | 35m (normal vs. sleep deprivation) | 0.658 | 0.176 | 0.420 | -0.165 | 1.481 |
| r=0.362 | Baati et al., 2020 | 15m (normal vs. first half of sleep deprivation) | 0.358 | 0.115 | 0.339 | -0.307 | 1.022 |
|  | Baati et al., 2020 | 25m (normal vs. first half of sleep deprivation) | 0.295 | 0.112 | 0.335 | -0.362 | 0.951 |
|  | Baati et al., 2020 | 35m (normal vs. first half of sleep deprivation) | 0.903 | 0.159 | 0.398 | 0.122 | 1.684 |
|  | Baati et al., 2020 | 15m (normal vs. second half of sleep deprivation) | 0.385 | 0.116 | 0.341 | -0.283 | 1.053 |
|  | Baati et al., 2020 | 25m (normal vs. second half of sleep deprivation) | 0.289 | 0.112 | 0.335 | -0.367 | 0.945 |
|  | Baati et al., 2020 | 35m (normal vs. second half of sleep deprivation) | 0.767 | 0.144 | 0.380 | 0.022 | 1.511 |
|  | Baati et al., 2015 | 15m (normal vs. sleep deprivation) | 0.422 | 0.118 | 0.344 | -0.252 | 1.095 |
|  | Baati *et al.,* 2015 | 25m (normal vs. sleep deprivation) | 0.626 | 0.132 | 0.363 | -0.085 | 1.338 |
|  | Baati *et al.,* 2015 | 35m (normal vs. sleep deprivation) | 0.642 | 0.133 | 0.365 | -0.073 | 1.357 |

Table 4: Verbal distance estimation pre- and post-exercise in athletes

|  | Paper | Measure | Hedge's g | Variance | SE | LL CI | UL CI |
| --- | --- | --- | --- | --- | --- | --- | --- |
| r=0.262 | Jarraya *et al.,* 2013 | Athletes group 5m (pre - post) | 10.079 | 4.239 | 2.059 | 6.043 | 14.114 |
|  | Jarraya *et al.,* 2013 | Athletes group 7m (pre - post) | 8.315 | 2.910 | 1.706 | 4.972 | 11.658 |
|  | Jarraya *et al.,* 2013 | Athletes group 9m (pre - post) | 8.716 | 3.190 | 1.786 | 5.216 | 12.217 |
|  | Jarraya *et al.,* 2013 | Athletes group 11m (pre - post) | 9.915 | 4.106 | 2.026 | 5.944 | 13.886 |
| r=0.362 | Jarraya *et al.,* 2013 | Athletes group 5m (pre - post) | 10.074 | 3.662 | 1.914 | 6.323 | 13.824 |
|  | Jarraya *et al.,* 2013 | Athletes group 7m (pre - post) | 8.299 | 2.506 | 1.583 | 5.196 | 11.401 |
|  | Jarraya *et al.,* 2013 | Athletes group 9m (pre - post) | 8.707 | 2.752 | 1.659 | 5.456 | 11.959 |
|  | Jarraya *et al.,* 2013 | Athletes group 11m (pre - post) | 9.890 | 3.531 | 1.879 | 6.207 | 13.573 |
| r=0.162 | Jarraya *et al.,* 2013 | Athletes group 5m (pre - post) | 10.082 | 4.817 | 2.195 | 5.780 | 14.384 |
|  | Jarraya *et al.,* 2013 | Athletes group 7m (pre - post) | 8.327 | 3.313 | 1.820 | 4.760 | 11.895 |
|  | Jarraya *et al.,* 2013 | Athletes group 9m (pre - post) | 8.723 | 3.628 | 1.905 | 4.990 | 12.456 |
|  | Jarraya *et al.,* 2013 | Athletes group 11m (pre - post) | 9.935 | 4.680 | 2.163 | 5.695 | 14.175 |

Table 5: Verbal hill steepness estimation pre- and post-exercise

|  | Paper | Measure | Hedge's g | Variance | SE | LL CI | UL CI |
| --- | --- | --- | --- | --- | --- | --- | --- |
| r = 0.35 | Bhalla and Proffitt Experiment 2, 1999 | 5deg (pre and post) | -0.789 | 0.080 | 0.283 | -1.344 | -0.234 |
|  | Bhalla and Proffitt Experiment 2, 1999 | 31deg (pre and post) | -0.915 | 0.087 | 0.295 | -1.494 | -0.337 |
|  | Proffitt *et al.*  Experiment 5, 1995 | 5deg (pre and post) | -1.039 | 0.077 | 0.277 | -1.583 | -0.495 |
|  | Proffitt *et al.*  Experiment 5, 1995 | 31deg (pre and post) | -0.884 | 0.070 | 0.264 | -1.401 | -0.367 |
| r=0.25 | Bhalla and Proffitt Experiment 2, 1999 | 5deg (pre and post) | -0.790 | 0.093 | 0.304 | -1.386 | -0.194 |
|  | Bhalla and Proffitt Experiment 2, 1999 | 31deg (pre and post) | -0.916 | 0.101 | 0.317 | -1.538 | -0.295 |
|  | Proffitt *et al.*  Experiment 5, 1995 | 5deg (pre and post) | -1.040 | 0.089 | 0.298 | -1.625 | -0.456 |
|  | Proffitt *et al.*  Experiment 5, 1995 | 31deg (pre and post) | -0.902 | 0.081 | 0.285 | -1.461 | -0.344 |
| r=0.45 | Bhalla and Proffitt Experiment 2, 1999 | 5deg (pre and post) | -0.788 | 0.068 | 0.260 | -1.298 | -0.278 |
|  | Bhalla and Proffitt Experiment 2, 1999 | 31deg (pre and post) | -0.914 | 0.074 | 0.271 | -1.446 | -0.382 |
|  | Proffitt *et al.*  Experiment 5, 1995 | 5deg (pre and post) | -1.038 | 0.065 | 0.255 | -1.538 | -0.538 |
|  | Proffitt *et al.*  Experiment 5, 1995 | 31deg (pre and post) | -0.861 | 0.058 | 0.241 | -1.333 | -0.389 |

Table 6: haptic hill steepness estimation pre- and post-exercise

|  | Paper | Measure | Hedge's g | Variance | SE | LL CI | UL CI |
| --- | --- | --- | --- | --- | --- | --- | --- |
| r= 0.35 | Bhalla and Proffitt Experiment 2, 1999 | 5deg (pre and post) | -0.371 | 0.064 | 0.254 | -0.869 | 0.126 |
|  | Bhalla and Proffitt Experiment 2, 1999 | 31deg (pre and post) | -0.055 | 0.060 | 0.245 | -0.535 | 0.425 |
|  | Proffitt *et al.*  Experiment 5, 1995 | 5deg (pre and post) | -0.385 | 0.054 | 0.232 | -0.839 | 0.069 |
|  | Proffitt *et al.*  Experiment 5, 1995 | 31deg (pre and post) | 0.164 | 0.051 | 0.225 | -0.277 | 0.605 |
| r= 0.25 | Bhalla and Proffitt Experiment 2, 1999 | 5deg (pre and post) | -0.373 | 0.074 | 0.273 | -0.907 | 0.161 |
|  | Bhalla and Proffitt Experiment 2, 1999 | 31deg (pre and post) | -0.055 | 0.069 | 0.263 | -0.571 | 0.461 |
|  | Proffitt *et al.*  Experiment 5, 1995 | 5deg (pre and post) | -0.385 | 0.062 | 0.249 | -0.873 | 0.103 |
|  | Proffitt *et al.*  Experiment 5, 1995 | 31deg (pre and post) | 0.167 | 0.059 | 0.242 | -0.307 | 0.641 |
| r= 0.45 | Bhalla and Proffitt Experiment 2, 1999 | 5deg (pre and post) | -0.369 | 0.054 | 0.233 | -0.826 | 0.088 |
|  | Bhalla and Proffitt Experiment 2, 1999 | 31deg (pre and post) | -0.054 | 0.051 | 0.225 | -0.496 | 0.387 |
|  | Proffitt *et al.*  Experiment 5, 1995 | 5deg (pre and post) | -0.384 | 0.045 | 0.213 | -0.802 | 0.033 |
|  | Proffitt *et al.*  Experiment 5, 1995 | 31deg (pre and post) | 0.159 | 0.043 | 0.207 | -0.247 | 0.565 |

Table 7: Verbal aperture distance estimation in body size manipulation

|  | Paper | Measure | Hedge's g | Variance | SE | LL CI | UL CI |
| --- | --- | --- | --- | --- | --- | --- | --- |
| r=0.35 | Collier Chapter 5, Experiment 1 | Padded vs. unpadded - intention to act | 0.003 | 0.066 | 0.257 | -0.500 | 0.506 |
|  | Collier Chapter 5, Experiment 2 | Padded vs. unpadded - R hand | 0.041 | 0.066 | 0.257 | -0.462 | 0.545 |
|  | Collier Chapter 5, Experiment 2 | Padded vs. unpadded - L hand | 0.011 | 0.066 | 0.257 | -0.493 | 0.514 |
| r=0.25 | Collier Chapter 5, Experiment 1 | Padded vs. unpadded - intention to act | 0.003 | 0.076 | 0.276 | -0.537 | 0.544 |
|  | Collier Chapter 5, Experiment 2 | Padded vs. unpadded - R hand | 0.041 | 0.076 | 0.276 | -0.500 | 0.582 |
|  | Collier Chapter 5, Experiment 2 | Padded vs. unpadded - L hand | 0.011 | 0.076 | 0.276 | -0.530 | 0.551 |
| r=0.45 | Collier Chapter 5, Experiment 1 | Padded vs. unpadded - intention to act | 0.003 | 0.056 | 0.236 | -0.460 | 0.466 |
|  | Collier Chapter 5, Experiment 2 | Padded vs. unpadded - R hand | 0.041 | 0.056 | 0.236 | -0.422 | 0.504 |
|  | Collier Chapter 5, Experiment 2 | Padded vs. unpadded - L hand | 0.011 | 0.056 | 0.236 | -0.452 | 0.474 |

Table 8: Distance estimation via blind-walking under conditions of external load

|  | Paper | Measure | Hedge's g | Variance | SE | LL CI | UL CI |
| --- | --- | --- | --- | --- | --- | --- | --- |
| r= 0.382 | Corlett *et al.* | light resistance group: condition 1(no resistance) vs. condition 2 (resistance) | -0.159 | 0.124 | 0.352 | -0.849 | 0.531 |
| r= 0.494 | Corlett *et al.* | heavy resistance group: condition 1(no resistance) vs. condition 2 (resistance) | -0.400 | 0.110 | 0.332 | -1.050 | 0.250 |
| r= 0.282 | Corlett *et al.* | light resistance group: condition 1(no resistance) vs. condition 2 (resistance) | -0.160 | 0.144 | 0.380 | -0.905 | 0.584 |
| r= 0.394 | Corlett *et al.* | heavy resistance group: condition 1(no resistance) vs. condition 2 (resistance) | -0.402 | 0.132 | 0.363 | -1.114 | 0.310 |
| r= 0.482 | Corlett *et al.* | light resistance group: condition 1(no resistance) vs. condition 2 (resistance) | -0.157 | 0.104 | 0.322 | -0.789 | 0.474 |
| r= 0.594 | Corlett *et al.* | heavy resistance group: condition 1(no resistance) vs. condition 2 (resistance) | -0.397 | 0.088 | 0.297 | -0.979 | 0.185 |

Table 9: Distance estimation via VR paradigm under conditions of external loads

|  | Paper | Measure | Hedge's g | Variance | SE | LL CI | UL CI |
| --- | --- | --- | --- | --- | --- | --- | --- |
| r= 0.382 | White experiment 1a | Control vs. backpack (10% body weight) | -0.055 | 0.052 | 0.229 | -0.503 | 0.393 |
| r= 0.494 | White experiment 1a | Control vs. ankle weights (10% body weight) | -0.643 | 0.052 | 0.229 | -1.091 | -0.195 |
| r= 0.282 | White experiment 1a | Control vs. backpack (10% body weight) | -0.055 | 0.061 | 0.246 | -0.538 | 0.428 |
| r= 0.394 | White experiment 1a | Control vs. ankle weights (10% body weight) | -0.646 | 0.063 | 0.250 | -1.136 | -0.155 |
| r= 0.482 | White experiment 1a | Control vs. backpack (10% body weight) | -0.055 | 0.044 | 0.209 | -0.465 | 0.356 |
| r= 0.594 | White experiment 1a | Control vs. ankle weights (10% body weight) | -0.639 | 0.042 | 0.205 | -1.040 | -0.238 |
