# Supplementary material for: Does who I am and what I feel determine what I see (or say)? A meta-analytic systematic review exploring the influence of real and perceived bodily state on spatial perception of the external environment
Source: PeerJ. 2022 May 23;10:e13383. doi: 10.7717/peerj.13383 (PMC9135041; doi:10.7717/peerj.13383)
Supplement: Supplemental Information 6 [file peerj-10-13383-s006.docx]

Supplementary 6: Contour enhanced funnel plots, for all the dark grey represents p < 0.1, mid grey p < 0.05, and the light grey p <0.01.
