# Supplementary material for: Does who I am and what I feel determine what I see (or say)? A meta-analytic systematic review exploring the influence of real and perceived bodily state on spatial perception of the external environment
Source: PeerJ. 2022 May 23;10:e13383. doi: 10.7717/peerj.13383 (PMC9135041; doi:10.7717/peerj.13383)
Supplement: Supplemental Information 7 [file peerj-10-13383-s007.docx]

1. **The rationale for conducting the systematic review/meta-analysis**

That the physiological and physical state of the body can influence visuospatial perception of the environment – so-called ‘embodied perception’ – has been countered with the idea that perceptual shifts represent a confound of experimental task demands and/or response bias. However, the literature in this field has never been comprehensively reviewed nor has it been critically appraised. Such appraisal across the field is important because, beyond task demands and response bias, many methodological features of a study can increase risk of bias, and thus the chance that the results are spurious. Additionally, assessment of the presence for publication bias is critical given that null findings may be less likely to be publishable in this area.

Therefore, we aimed to undertake a systematic review and meta-analysis to appraise the evidence regarding the effect of bodily state (objective bodily state and subjective perceptions about body state) on visuospatial perception of the external environment.

1. **The contribution that it makes to knowledge in light of previously published related reports, including other meta-analysis and systematic reviews**

Our findings suggest that high methodological bias present within the literature of this field make any conclusions about reported effects reflecting true perceptual shifts, rather than experimental task demands, pre-emptive. That is, higher quality research to exclude other forms of methodological bias is needed before the contribution of experimental task demands to the effects seen can be clarified. This extends the findings of a past review (Molto 2020) that specifically focussed on the influence of external action-constraints (e.g., tool use) on visuospatial perception. This previous meta-analysis assessed whether experimental demand bias was present through a moderator analysis. They found no support for the influence of experimental design (between vs. within group), or type of spatial perception measure (verbal vs. visual or action-based) on observed effect sizes. However, we contend, given the high risk of bias across multiple domains, such comparisons may be premature. The null results of the moderator analysis may reflect high variability of effects due to high risk of bias across multiple domains, rather than a true null result.

Despite these limitations, our systematic review and meta-analysis found consistent evidence that age and blood glucose levels influence spatial perception in line with proposed perceptual shifts relate to bodily capacity (where reduced bodily capacity is associated with increased perceived distance and hill steepness). There was mixed evidence for changes in visuospatial perception based upon body size/body part size, embodiment, interoceptive accuracy, pain, and external loads. There were conflicting results for the effect of fitness and of fatigue on visuospatial perception of the environment, but methodological flaws with fitness and fatigue paradigms may underlie null/conflicting results. Evidence for publication bias was present for studies evaluating the effect of age on visuospatial perception, but was unable to be reliably assessed for the remaining bodily state groupings, given low study numbers.

Based on our risk of bias assessment we make field specific recommendations to enhance methodological rigour. Namely, the present literature would be improved by methodologically (or statistically) controlling for known confounders, using valid and reliable bodily state manipulation paradigms (including assessment of manipulation success/failure), establishing reliability of visuospatial perception measures and, ensuring both blinded assessment of visuospatial perception and blinded completion of statistical analysis (i.e., by an investigator to whom group/condition status is unknown).
